# Effectiveness of cognitive behavioral therapy for depression in patients with multiple sclerosis: A systematic review and meta-analysis

**DOI:** 10.1097/MD.0000000000048327

**Published:** 2026-04-17

**Authors:** Wenli Xu, Bin Shen, Huali Xu, Yaoying Zhou, Yefeng Wang

**Affiliations:** a Nursing Department, Shaoxing People’s Hospital, Shaoxing, Zhejiang, China.

**Keywords:** cognitive-behavior therapy, depressive symptom, multiple sclerosis, psychotherapy

## Abstract

**Background:**

The efficacy and methodologies of cognitive behavioral therapy (CBT) for treating depression in multiple sclerosis (MS) require further comprehensive research.

**Methods:**

Two researchers conducted independent literature searches across 5 databases, utilizing the keywords “multiple sclerosis,” “cognitive behavioral therapy,” and “depression,” without any restrictions on publication year. The Cochrane Risk of Bias Tool 2.0 was utilized to assess the included studies. Statistical analyses were conducted using standardized mean difference as the effect size with Stata 17.0 software.

**Results:**

This meta-analysis included 22 studies. The results indicate that, regardless of whether delivered face-to-face, online, or via telephone, both individual and group CBT effectively alleviate depression in patients with MS. Significant improvements in depressive symptoms were observed starting from the second month of intervention and persisted for up to 6 months post-intervention.

**Conclusion:**

Patients with MS can flexibly choose the delivery modality of CBT based on their individual circumstances while engaging in treatment for around 2 months. They are encouraged to reengage in CBT within 6 months post-intervention to ensure the continuity and stability of depression treatment outcomes. Healthcare providers can offer various CBT delivery modalities based on the individual preferences and circumstances of patients with MS, while developing corresponding plans to enhance the effectiveness of depression management.

## 1. Background

Multiple sclerosis (MS) is an immune-mediated chronic inflammation of the central nervous system. In addition to its direct impact on the nervous system, it can also lead to a range of neuro-psychiatric symptoms, such as depression, fatigue, and cognitive impairment.^[1]^ Globally, approximately 2.8 million individuals are impacted by MS, with patients typically being under the age of 50.^[2]^ A cure for the disease is not currently known, and the existing treatment options, primarily immunotherapies aimed at curbing disease progression, are limited and come with notable side effects.^[3]^ Of particular concern is the potential manifestation of depression as an early symptom in individuals with MS, with both its incidence and severity often escalating with disease progression.^[4]^

According to statistics, the prevalence of severe depression within 12 months among MS patients can be as high as 25%,^[5]^ with a lifetime prevalence reaching up to 50%.^[6]^ If depressive symptoms are not effectively managed, they are seldom spontaneously alleviated and can evolve into chronic manifestations, progressively worsening over time.^[7,8]^ This not only disrupts patients’ interpersonal relationships and professional lives but also diminishes medication adherence and overall quality of life, emerging as a prominent predictor of suicidal thoughts and suicide risk in MS patients.^[9–11]^ Therefore, enhancing awareness of the psychological well-being of MS patients and providing tailored support is paramount.

Despite the direct clinical significance of depressive symptoms in individuals with MS, the diagnosis and treatment of depression in this population generally fall short.^[12]^ Presently, the preferred treatment for improving depressive symptoms is pharmacotherapy, specifically the use of antidepressant medications managed by healthcare professionals.^[13,14]^ However, evidence regarding the efficacy of this treatment in patients with MS is insufficient.^[13,14]^ Conversely, a substantial body of research indicates the potential benefits of cognitive behavioral therapy (CBT) in mitigating depressive symptoms among MS patients.^[15,16]^ The fundamental principle of CBT is to aid individuals in identifying and modifying negative thought patterns and behaviors, thereby promoting positive emotional and behavioral outcomes. Despite these promising initial findings, the American Academy of Neurology emphasizes the necessity for further research, particularly in the development and rigorous testing of treatment strategies for depression specifically related to MS, with a focus on enhancing their practical feasibility.^[14]^

In comparison to traditional face-to-face CBT, delivering therapy via telephone assists patients in overcoming treatment barriers such as limited mobility and fatigue, offering flexible options that allow them to receive therapy comfortably at home, thereby mitigating transportation and geographical constraints.^[17]^ Nonetheless, a significant challenge lies in the scarcity of therapists capable of meeting the demand for CBT and other psychological treatments among patients, which may lead to insufficient medical resources and extended waiting times.^[18]^ Additionally, telephone CBT has limitations in information transfer; the absence of nonverbal cues may hinder the therapist’s understanding of the patient’s emotional state, potentially affecting the effectiveness of the therapy. In contrast, remote access methods, particularly online psychological tools, typically provide a wealth of learning materials, videos, and interactive tools, allowing patients to engage in personalized learning based on their needs. This accessibility is not constrained by therapist availability, making it more universally applicable than telephone CBT.^[19,20]^ However, there is a concern that online CBT often requires patients to have stronger self-management skills, and some may discontinue treatment due to a lack of supervision.^[21]^

CBT not only exhibits diverse delivery methods, but the differences in effectiveness between individual and group treatments have also been a topic of significant debate. The advantage of individual CBT lies in its personalized approach, allowing therapists to create tailored treatment plans based on the specific issues of each patient, thus providing a higher sense of privacy and security.^[22]^ Furthermore, the flexibility of individual therapy allows for timely adjustments according to the patient’s progress.^[23]^ However, the higher costs associated with individual therapy may pose a barrier for some patients, and the lack of social support can lead to feelings of isolation during treatment.^[24,25]^ In contrast, group CBT offers clear advantages in terms of social interaction and shared experiences. Patients can engage with one another, gain empathy and encouragement, and group therapy is typically more cost-effective, making it more accessible to a larger number of individuals. Nonetheless, group therapy may also face challenges related to reduced individual attention; the therapist’s divided focus may not adequately address the specific needs of each patient, and some individuals may feel uncomfortable in a group setting.^[26]^ Therefore, it is crucial to clarify the effectiveness of individual and group CBT in treating depression in patients with MS. This will provide patients with the opportunity to freely choose a treatment approach that best suits their circumstances, ultimately optimizing their treatment experience and outcomes.

In light of this context, the objective of our meta-analysis is to thoroughly review and update recent studies regarding the effect of CBT on depression symptoms in MS patients. Simultaneously, we seek to investigate effective and feasible implementation strategies. The objective is to provide guidance for the clinical implementation of CBT for MS patients experiencing comorbid depression.

## 2. Methods

The review adheres to the methodology outlined in the Cochrane Handbook for Systematic Reviews of Interventions and follows the reporting guidelines outlined in the Preferred Reporting Items for Systematic Reviews and Meta-Analyses reporting guidelines.^[27]^ This study is prospectively registered in PROSPERO under the registration number CRD42023484280.

### 2.1. Search strategy

We conducted a thorough search of multiple databases from their inception to April 2024, including MEDLINE, EMBASE, SCOPUS, Cochrane, and PsycINFO. Our search strategy was inspired by the existing reviews, incorporating search terms related to MS, CBT, and depression.^[15,16]^ A careful screening of relevant references and gray literature was conducted simultaneously to ensure the comprehensiveness of the search.

### 2.2. Eligibility criteria

In order to define the research question, we formulated specific inclusion and exclusion criteria using the PICOS (Participants, Interventions, Comparisons, Outcome, and Study design) strategy.^[28]^ The inclusion criteria were: (P) Participants: patients aged 18 years and older who have been diagnosed with MS and depression; (I) Interventions: cognitive behavioral therapy; (C) Comparisons: waiting list control, standard care, usual care, or no treatment for depression; (O) Outcomes: depression; (S) Study design: randomized controlled trial (RCT) and quasi-RCTs. The exclusion criteria included: articles published in languages other than English; conference abstracts; duplicate publications; studies with incomplete data; and studies rated as “high risk” for quality.

### 2.3. Study selection

The electronic literature search was conducted by a single reviewer, who imported all identified records into Endnote software (Camelot UK Bidco Limited, UK) for efficient management and processing. To ensure a uniform understanding and consistent application of the PICO selection criteria during the review process, all reviewers participated in preliminary training aimed at enhancing their evaluation skills and expertise. Subsequently, 2 reviewers independently screened the titles and abstracts to ensure no relevant literature was overlooked. In cases of any discrepancies, the reviewers engaged in discussions to reach a consensus, and a third reviewer was consulted when necessary to ensure the accuracy and reliability of the review outcomes.

### 2.4. Data extraction

The form included crucial details such as the first author’s name, nationality, study design, participant demographics (e.g., age, gender, and sample size), intervention implementation specifics, and the depression rating scales. Furthermore, to comprehensively evaluate the effectiveness of the interventions, we extracted depression scores from each study at any point during intervention, at the cessation of intervention, and within the subsequent year. Any discrepancies in decision-making were resolved by a third researcher, ensuring objectivity during the data extraction process. When necessary, comprehensive datasets were obtained through email correspondence with the respective corresponding authors.

### 2.5. Evaluation of risk of bias

Two researchers utilized the Cochrane Risk of Bias Tool 2.0^[29]^ to systematically assess the treatments included in the study, covering 6 domains: randomization process, deviation from the intended intervention, missing outcome data, outcome measurement, selective reporting of results, and overall bias. In instances of divergence during the evaluation, the researchers engaged in discussions with a third peer to address the discrepancies in outcomes. Each item was categorized as low, high, or unclear, ensuring a comprehensive and nuanced assessment.

### 2.6. Data synthesis and publication bias

Given that the outcome measures in this study are continuous variables, we used mean ± standard deviation as the effect size and conducted double verification to ensure data accuracy. When a study utilized multiple depression scales, we prioritized the most commonly used ones. Statistical analysis was performed using a random effects model in Stata 17 (StataCorp LLC, College Station), synthesizing the data through SMD, where an SMD <0 indicates that the average depression score in the CBT group is lower than that in the control group, suggesting that CBT is more favorable. Additionally, we quantified the heterogeneity among studies using the *I*^2^ statistic and *Q* test to comprehensively assess the impact of statistical heterogeneity on the results.^[30]^ Statistical significance was set at a two-tailed *P* < .05. To assess publication bias, we employed a funnel plot and conducted quantitative analyses using Egger and Begg tests.^[31–33]^

### 2.7. Sensitivity analysis

Sensitivity analysis was conducted to assess the stability of the results by excluding studies with a high degree of overall bias or excluding individual studies.^[34]^ This approach aimed to investigate the sources of heterogeneity and ensure the robustness of the findings.

### 2.8. Subgroup

The main results involved analyzing CBT based on time, including differences in effects during the intervention, at the end of the intervention, and within 12 months post-intervention, to explore the relationship between CBT and intervention timing. Additionally, subgroup analyses covered various factors such as modes of delivery, individual/group format, duration of intervention, and country to investigate differences in the effectiveness of CBT for depression in MS patients.

## 3. Results

### 3.1. Search process

**Figure 1.**
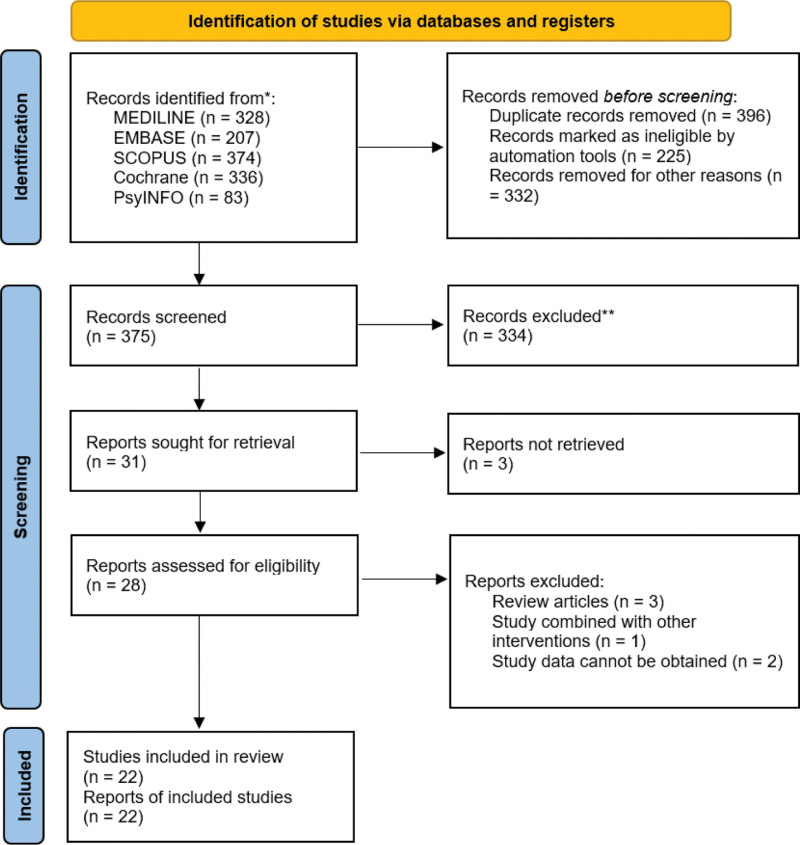
PRISMA flowchart showing the study screening and selection process. PRISMA = Preferred Reporting Items for Systematic Reviews and Meta-Analyses.

This study reviewed a total of 1328 articles, of which 31 were selected for abstract reading after initial screening based on titles. Among these, 28 abstracts were further subjected to full-text review. Additionally, 2 articles were included following manual searches. Ultimately, 22 studies^[35–56]^ were incorporated into this review, involving a total of 1558 participants. The detailed literature screening process is shown in Figure 1.

### 3.2. Trial characteristics

Between 2000 and 2023, a total of 22 studies were conducted, comprising 6 in the USA,^[45,48–50,53,54]^ 6 in the UK,^[36,38,40,47,51,56]^ 3 in Iran,^[35,42,52]^ and 2 each in Italy^[37,44]^ and Germany.^[39,43]^ Additionally, there was one study each in Australia,^[46]^ Netherlands,^[55]^ and France.^[41]^ Among these trials, 20^[35,36,38–41,43–56]^ were RCTs, and 2^[37,42]^ were quasi-experimental trials. The studies involved a collective total of 1558 participants, with the intervention group’s age ranging from 34.60 to 53.42 years, totaling 832 individuals, and the control group’s age ranging from 34.8 to 53 years, totaling 726 individuals. The intervention formats included online, telephone, and face-to-face sessions, conducted either individually or in group settings. Each intervention session varied in duration from 30 to 120 minutes, and the intervention periods spanned from 6 to 16 weeks. Further details on the studies’ specifics, participant demographics, intervention details, and depression assessment scales are accessible in Table 1.

**Table 1:** Summary of sample characteristics, interventions, and outcome rating scales in included studies.

References	Country	Study	Simple sizes (M/F)		Age (yr)			Intervention (CBT)			Comparator	Depression assessment scale
			IG	CG	IG	CG	Modes of delivery	Individual/group	Course length/frequency	Total duration		
Gold et al^[43]^	Germany	RCT	G1: 95 (23/72) G2: 79 (20/59)	88 (19/69)	G1: 46.5 ± 11.9 G2: 47.1 ± 12.1	47.3 ± 11.1	Online	Individual	30–60 min/once a module	12 wk	Usual care	BDI
Gay et al^[41]^	France	RCT	57 (7/50)	48 (9/39)	43.2 ± 9.4	47.4 ± 10.0	Face to face	Group	90-min/weekly	6 wk	Standard care	HADS
Sadeghi-Bahmani et al^[53]^	USA	RCT	26 (3/23)	25 (7/18)	37.21 ± 9.83	40.20 ± 10.72	Face to face	Group	90–120 min/weekly	8 wk	Waitlist	BDI
Siengsukon et al^[54]^	USA	RCT	10 (1/9)	10 (2/8)	51.1 ± 7.9	50.4 ± 12.4	Face to face	Individual	30-45 min/weekly	6 wk	Usual care	PHQ-9
Gromisch et al^[45]^	USA	RCT	10 (6/4)	10 (6/4)	52.20 ± 9.61	53.00 ± 12.66	Face to face + telephone	Individual	Seven 1-hr sessions + five 30-min telephone sessions/NR	15 wk	Standard care	BDI
Ghodspour et al^[42]^	Iran	QET	15 (0/15)	15 (0/15)	36 ± 6	36 ± 6	NR	NR	2-hr/weekly	8 wk	No treatment	DASS
van den Akker et al^[55]^	The Netherlands	RCT	44 (13/31)	47 (8/39)	50.6 ± 8.3	46.4 ± 11.6	Face to face	Individual	8 sessions in the first 2 mo + 4 sessions in the last 2 mo/NR	16 wk	Usual care	HADS
Bahrani et al^[35]^	Iran	RCT	28	28	36.78 ± 6.12	36 ± 7.08	Face to face	Group	2-h/weekly	8 wk	Usual care	DASS
Calandri et al^[37]^	Italy	QET	54 (21/33)	31 (14/17)	38 ± 12.5	34.8 ± 11.9	Face to face	Group	Five 2-h sessions/NR	8 wk	Usual care	CESD-10
Pahlavanzadeh et al^[52]^	Iran	RCT	35 (0/35)	35 (0/35)	NR	NR	Face to face	Group	90-min/weekly	8 weeks	Usual care	DASS
Fischer et al^[39]^	Germany	RCT	45 (11/34)	45 (9/36)	45.36 ± 12.64	45.2 ± 10.56	Online	Individual	<60 min sessions/once a module	9 wk	Standard care	BDI
Kiropoulos et al^[46]^	Australia	RCT	15 (2/13)	15 (6/9)	34.60 ± 9.6	39.27 ± 9.93	Face to face	Individual	60 min/once a session	8 wk	Standard care	BDI
Bogosian et al^[36]^	UK	RCT	19 (10/9)	21 (8/13)	53.42 ± 8.3	50.9 ± 9.9	Online	Group	60 min sessions/once a session	8 wk	Usual care	HADS
Graziano et al^[44]^	Italy	RCT	41 (14/27)	41 (17/24)	42.3 ± 8.5	38.3 ± 10.1	Face to face	Group	Four 2-h sessions + 2-h session after 6 mo/NR	over 8 wk	Usual care	CESD-10
Moss-Morris et al^[51]^	UK	RCT	23 (7/16)	17 (1/16)	40.14 ± 17.76	41.8 ± 11.43	Online	Individual	8 sessions + three 30–60 min telephone sessions/weekly	8–10 wk	Standard care	HADS
Cooper et al^[38]^	UK	RCT	12 (1/11)	12 (5/7)	48.0 ± 7.7	42 ± 7.0	Online	Individual	50-min/weekly	8 wk	Usual care	BDI, PHQ-9
Lincoln et al^[47]^	UK	RCT	72	79	44.5 ± 11.1	47.5 ± 10.5	Face to face	Group	Six 2-hour sessions/NR	12 wk	Waitlist	BDI, HADS
Forman and Lincoln^[40]^	UK	RCT	19	19	47.3 ± 10.3	47.7 ± 9.8	Face to face	Group	Six 2-h sessions/biweekly	12 wk	Waitlist	HADS
van Kessel et al^[56]^	UK	RCT	35 (7/28)	37 (10/27)	42.89 ± 9.29	47.03 ± 9.45	Face to face + telephone	Individual	Three 50-min face-to-face sessions + five 50-min telephone sessions/weekly	8 wk	Standard care	HADS
Mohr et al^[49]^	USA	RCT	62 (15/47)	65 (14/51)	48.60 ± 9.62	47.3 ± 10.1	Telephone	Individual	50-min sessions/weekly	16 wk	Usual care	BDI, HRSD
Mohr et al^[48]^	USA	RCT	20	22	43.90 ± 10.00	43.9 ± 10.0	Face to face	Individual	50-min sessions/weekly	16 wk	Usual care	BDI, HRSD
Mohr et al^[50]^	USA	RCT	16 (6/10)	16 (3/13)	42.6 ± 12.8	42.1 ± 9.4	Telephone	Individual	50-min sessions/weekly	8 wk	Usual care	POMS

BDI = the Beck Depression Inventory, CBT = cognitive behavioral therapy, CESD-10 = the 10-item Center for Epidemiologic Studies Depression Scale, CG = control group, DASS = Depression Anxiety Stress Scales, G1 = Group 1 (stand-alone internet-based cognitive behavioral therapy), G2 = Group 2 (guided internet-based cognitive behavioral therapy), HADS = Hospital Anxiety and Depression Scale, HRSD = Hamilton Rating Scale for Depression, IG = intervention group, NR = not reported, PHQ-9 = Patient Health Questionnaire, POMS = profile of mood states, QET = quasi-experimental trial, RCT = randomized controlled trial.

### 3.3. Risk of bias assessment

**Figure 2.**
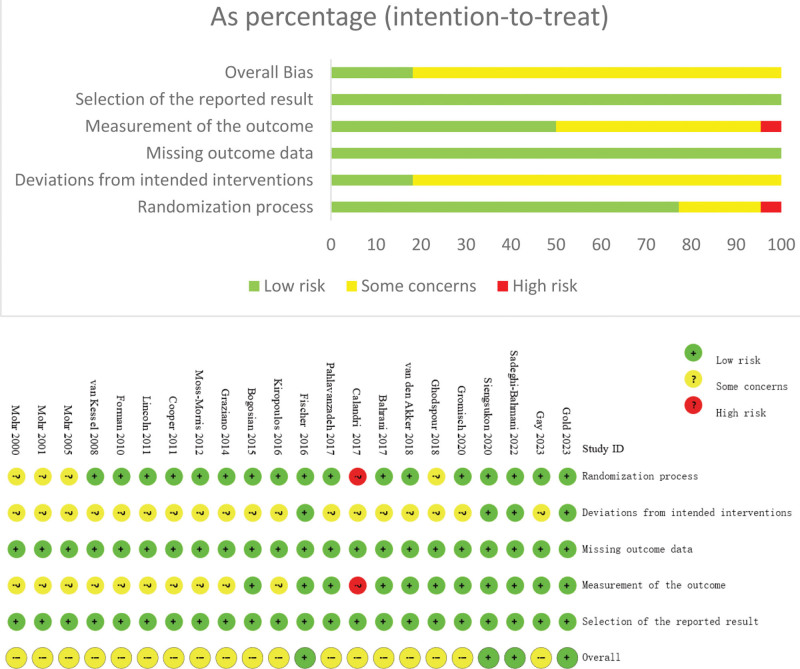
Methodological quality and risk of bias of the included studies. Qualitative assessment of the methodological quality items is presented as percentages across all included studies. Qualitative assessment of the risk-of-bias items.

As shown in Figure 2, the results of the Risk of Bias Tool 2.0 assessment indicate that 18 trials were rated as having “some concern,” while 4 trials were classified as having “low risk.” Among these trials, the primary reasons for the risk of bias included issues related to the randomization process (n = 4), deviations from the intended intervention (n = 19), and inadequacies in outcome measurement (n = 10).

### 3.4. Effects of CBT on depression in patients with MS

**Figure 3.**
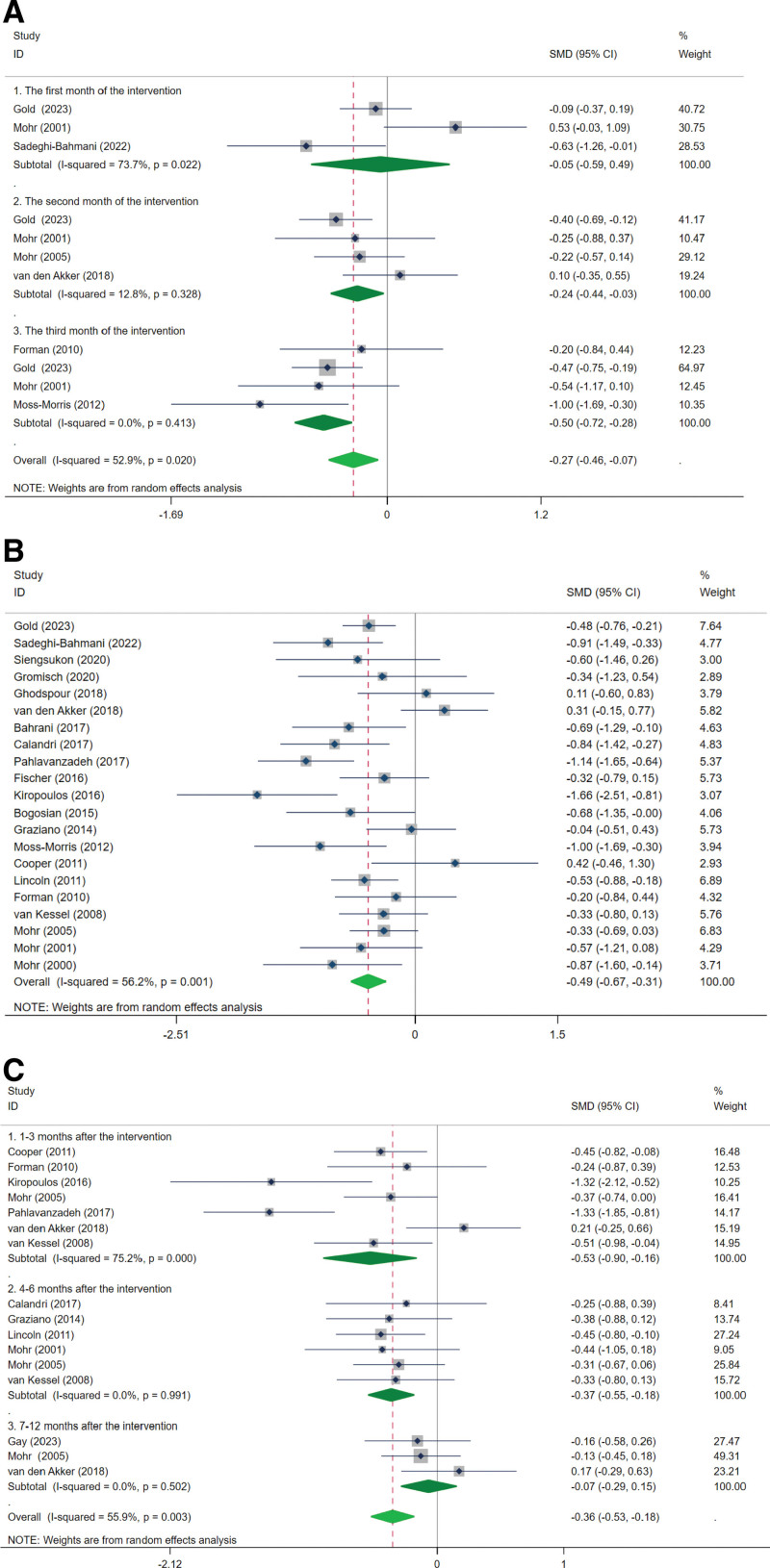
(A) The effect of CBT on depression in patients with multiple sclerosis during the intervention. (B) The effect of CBT on depression in patients with multiple sclerosis at the end of the intervention. (C) The effect of CBT on depression in patients with multiple sclerosis after the intervention. CBT = cognitive behavioral therapy.

Seven studies^[40,43,48,49,51,53,55]^ reported the effect of CBT on depression in MS patients during intervention. The analyses covered various intervention time points, including the 1st month,^[43,48,53]^ 2 months,^[43,48,49,55]^ and 3 months.^[40,43,48,51]^ Overall, the effects of CBT on depressive symptoms during the intervention were statistically significant (SMD, −0.27 [‐0.46, −0.07], *P* = .007; *I*^2^ = 52.9%; Fig. 3A). Specifically, in the 1st month, 3 studies showed no significant impact of CBT on depressive symptoms (SMD, −0.05 [‐0.59, 0.49], *P* = .846; *I*^2^ = 73.7%; Fig. 3A). In contrast, 4 studies indicated a significant improvement in depressive symptoms after a 2-month intervention (SMD, −0.24 [‐0.44, −0.03], *P* = .027; *I*^2^ = 12.8%; Fig. 3A). After 3 months, the effect of CBT on depression became statistically significant (SMD, −0.50 [‐0.72, −0.28], *P* < .0001; *I*^2^ = 0%; Fig. 3A).

At the conclusion of interventions, data from 21 studies^[35–40,42–56]^ revealed a notable improvement in depression among MS patients (SMD, −0.49 [‐0.67, −0.31], *P* < .0001; *I*^2^ = 56.2%; Fig. 3B). Additionally, 7 studies^[38,40,46,49,52,55,56]^ reported on depression status 1 to 3 months post-intervention, indicating continued positive effects of CBT (SMD, −0.53 [‐0.90, −0.16], *P* = .005; *I*^2^ = 75.2%; Fig. 3C). Six studies^[37,44,47–49,56]^ further demonstrated that the significant effect of CBT on depression persisted 4 to 6 months after the intervention (SMD, −0.37 [‐0.55, −0.18], *P* < .0001; *I*^2^ = 0%; Fig. 3C). However, 3 studies^[41,49,55]^ found no significant difference between the CBT and control groups 7 to 12 months post-intervention (SMD, −0.07 [‐0.29, 0.15], *P* = .541; *I*^2^ = 0%; Fig. 3C).

### 3.5. Sensitivity analysis

In the investigation of heterogeneity sources, a sensitivity analysis was conducted through systematic exclusion of individual studies. Our findings revealed that the exclusion of any single study had a minimal impact on the overall results. This indicates the relative stability of the combined effect size in our study.

### 3.6. Publication bias

The funnel plot exhibits a degree of symmetry, indicating comparable shapes, trends, or values on both sides. This observation substantiates the absence of conspicuous publication bias in the included studies. The uniform distribution enhances the credibility and interpretability of our research. Furthermore, for a comprehensive assessment of publication bias, we concurrently employed Egger and Begg tests, yielding results with *P* > .05. These tests further validated the quality of included literature, reinforcing our control over potential publication bias.

### 3.7. Subgroup analysis

**Figure 4.**
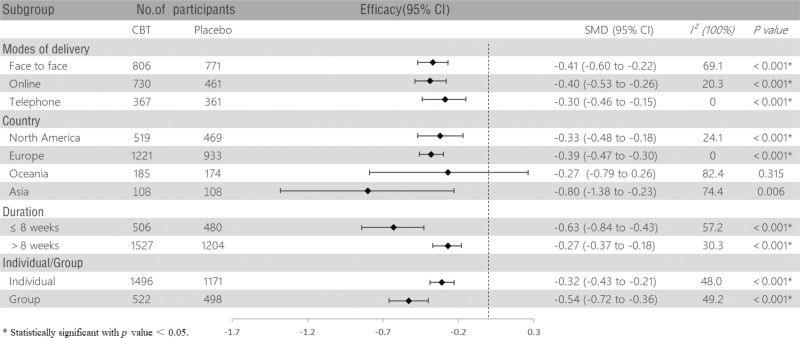
Subgroup analysis of the effects of CBT on depression in patients with multiple sclerosis. CBT = cognitive behavioral therapy.

The subgroup analysis based on delivery method showed that CBT, whether delivered face-to-face (SMD, −0.41 [‐0.60, −0.22], *P* < .001; *I*^2^ = 69.1%), via telephone (SMD, −0.30 [‐0.46, −0.15], *P* < .001; *I*^2^ = 0%), or online (SMD, −0.40 [‐0.53, −0.26], *P* < .001; *I*^2^ = 20.3%), has a significant positive impact on alleviating depression symptoms in patients with MS. The analysis regarding intervention duration indicated that both 6 to 8 weeks (SMD, −0.63 [‐0.84, −0.43], *P* < .001; *I*^2^ = 57.2%) and >8 weeks (SMD, −0.27 [‐0.37, −0.18], *P* < .001; *I*^2^ = 30.3%) of intervention were effective in improving patients’ depressive symptoms. Furthermore, there was no significant difference in effectiveness between individual CBT (SMD, −0.32 [‐0.43, −0.21], *P* < .001; *I*^2^ = 48.0%) and group CBT (SMD, −0.54 [‐0.72, −0.36], *P* < .001; *I*^2^ = 49.2%). In the subgroup analysis by country, results demonstrated that CBT was statistically significant for depressed patients in North America (SMD, −0.33 [‐0.48, −0.18], *P* < .001; *I*^2^ = 24.1%), Europe (SMD, −0.39 [‐0.47, −0.30], *P* < .001; *I*^2^ = 0%), and Asia (SMD, −0.80 [‐1.38, −0.23], *P* = .006; *I*^2^ = 74.4%), but no significant positive effects were observed among MS patients in Oceania (SMD, −0.27 [‐0.79, 0.26], *P* = .315; *I*^2^ = 82.4%). Detailed subgroup analyses are presented in Figure 4.

## 4. Discussion

According to our knowledge, our study represents the most extensive meta-analysis to date concerning the efficacy of CBT in alleviating depression among patients with MS, and this recommendation aims to serve as a reference for clinical practice, optimizing the efficacy of CBT in the treatment of depression among MS patients. Distinguishing itself from prior reviews,^[15,16]^ our study systematically investigates the impact of CBT interventions by considering intervention duration. Specifically, we evaluate outcomes at 1 to 3 months during intervention, at the conclusion of intervention, and 1-year post-intervention. Current findings indicate that any form of CBT, typically lasting between 6 weeks and 4 months, has a significant positive effect on the treatment of depression in MS patients. We observed that the effects of CBT interventions begin to manifest in the second month of treatment, but do not remain significant 6 months after the intervention concludes. Therefore, when designing CBT treatment plans for MS patients, therapists should consider extending the duration of the intervention to approximately 2 months. Furthermore, reinforcing CBT implementation within the 6 months following the intervention may help ensure more durable and stable treatment outcomes.

In addition to the duration of CBT interventions, the delivery methods of CBT have also become a focal point of research.^[19,20]^ Factors such as geographical distance, scarcity of qualified therapists, lengthy waiting lists, and economic constraints often hinder individuals from seeking traditional therapies, a challenge that is particularly pronounced among patients with MS.^[57–59]^ Online CBT offers an initial point of contact or a viable alternative to face-to-face treatment for those unable to access conventional therapy. The interaction between therapists and patients is facilitated through formats such as video conferencing, chat, email, or dedicated applications, making the therapeutic process closer to in-person experiences.^[60,61]^ Furthermore, online CBT facilitates the conversion of self-help materials into web-based modules, providing flexible treatment options; therapists need only invest minimal time in guiding patients on how to use these modules, thereby achieving effective therapeutic outcomes.^[62]^ Simultaneously, with the rise of mobile applications, traditional telephone interventions are shifting towards app-based interventions. This transition highlights the significant potential of digital technology in mental health interventions. Smartphone applications enable patients to receive support when symptoms arise, allowing them to flexibly choose intervention timings based on personal needs and daily life, thereby liberating them from fixed appointment constraints.^[63]^ Moreover, these applications often incorporate interactive features such as self-monitoring, real-time feedback, and community support, enhancing patients’ abilities to manage their symptoms effectively.^[64]^

Additionally, our findings suggest that group CBT can serve as a cost-effective alternative to individual CBT; however, the group format may lead to uneven progress among participants and reduce individual attention from therapists, while privacy concerns could hinder open communication within the group and increase avoidance behaviors.^[65]^ To address this challenge, online anonymous group CBT has emerged as a promising trend. It offers a supportive environment that allows participants to understand and encourage one another, sharing similar experiences to foster a sense of belonging and connection. However, as noted by Weinberg et al, current online community CBT has not received sufficient attention, and related research is relatively limited.^[66]^ It is believed that investing in research in this area can better support healthcare providers in delivering effective, flexible, and accessible mental health interventions in an increasingly digital world.

Based on the results of this study and from a broader perspective, we suggest that practitioners should actively promote the multimodal application of CBT to more effectively meet patient needs. Firstly, during patient consultations, healthcare professionals should engage in thorough communication to fully understand individual needs and expectations, allowing for the development of tailored participation plans.^[67]^ These plans should clearly outline participation goals, expected timelines, necessary resources, and integrate educational components to enhance patient awareness of new treatment modalities, effectively increasing their willingness to engage. Secondly, strategies for ongoing participation are equally important. Regular follow-ups and feedback mechanisms can help patients maintain a sense of belonging throughout the treatment process. Furthermore, establishing peer support groups can enhance mutual support and motivation among patients, representing a significant future direction.^[68]^ By implementing these comprehensive strategies, healthcare systems will more effectively meet the needs of MS patients, promote their mental health, and improve the overall efficiency and accessibility of mental health services.

In addition, from a policy perspective, to better meet the diverse treatment needs of the growing number of individuals with depression related to MS, healthcare policies should actively promote training and support for practitioners in emerging therapeutic methods. Specifically, policies should encourage therapists to participate in relevant training for digital psychotherapy and group therapy, enabling them to effectively establish trust in virtual environments, facilitate interaction and sharing among patients, and learn skills related to self-disclosure and empathy, thereby enhancing patients’ sense of participation and belonging.^[69]^ Moreover, to effectively control the therapeutic environment, policies should support training in relevant technologies, ensuring that practitioners are proficient in using online tools while creating a safe and supportive atmosphere.^[66]^ Through these policy measures, healthcare systems will be able to provide patients with more flexible and accessible treatment options, ultimately improving treatment efficacy and helping them better cope with the challenges posed by depression.

In conclusion, this review supports the multimodal implementation of CBT for the treatment of depression in MS patients through a comprehensive analysis, demonstrating CBT’s adaptability and inclusivity in addressing the complex needs of depressed MS patients. This finding is particularly significant for individuals with chronic conditions and represents a key highlight of our research.

In the subgroup analysis, despite the seemingly less significant impact of CBT on depression among MS patients in the Oceania region, we emphasize the potential uncertainty of this conclusion due to the limited sample size. This limitation underscores the need for future CBT research involving a more extensive representation of MS patients in Oceania to validate our findings. Such in-depth research would provide a comprehensive understanding of the unique requirements of patients in this region, offering more precise guidance to enhance the effectiveness of depression treatment services. Additionally, due to the variability in CBT course content and duration across studies, we were unable to conduct a detailed analysis of specific course formats, necessitating further research to provide more actionable clinical recommendations.

## 5. Conclusion

MS patients can opt for either group or individual CBT sessions lasting at least 2 months, depending on their specific circumstances. Whether conducted in-person, over the phone, or online, these approaches contribute to improving their depressive symptoms. Furthermore, reinforcing the implementation of CBT within 6 months following intervention may help ensure a more enduring and stable therapeutic effect.

## 6. Practice implications

Healthcare providers should consider offering diverse delivery modalities for CBT, tailored to the individual preferences and circumstances of patients with MS. By understanding each patient’s unique situation (such as mobility challenges, access to technology, and personal comfort) providers can create customized treatment plans that enhance engagement and adherence to therapy. Additionally, integrating regular follow-ups and feedback mechanisms will help monitor progress and make necessary adjustments, ultimately improving the effectiveness of depression management for patients with MS. This patient-centered approach not only fosters a supportive therapeutic environment but also empowers patients in their journey towards improved mental health.

## Author contributions

**Conceptualization:** Huali Xu.

**Data curation:** Wenli Xu, Huali Xu, Yaoying Zhou.

**Formal analysis:** Yefeng Wang.

**Software:** Wenli Xu, Bin Shen, Huali Xu, Yefeng Wang.

**Supervision:** Yefeng Wang.

**Writing – original draft:** Bin Shen.

**Writing – review & editing:** Huali Xu, Yefeng Wang.
